# Non-invasive 40-Hz Light Flicker Ameliorates Alzheimer’s-Associated Rhythm Disorder via Regulating Central Circadian Clock in Mice

**DOI:** 10.3389/fphys.2020.00294

**Published:** 2020-04-24

**Authors:** Youli Yao, Ying Ying, Qiyu Deng, Wenjiang Zhang, Huazhang Zhu, Zhenglong Lin, Shengli Zhang, Junxian Ma, Yingying Zhao

**Affiliations:** ^1^Department of Physiology, School of Basic Medical Sciences, Shenzhen University Health Science Center, Shenzhen University, Shenzhen, China; ^2^College of Electronics and Information Engineering, Shenzhen University, Shenzhen, China

**Keywords:** 40-Hz light flicker, Alzheimer’s disease, rhythm disorders, SCN neurons, circadian clock

## Abstract

Alzheimer’s disease (AD) patients often exhibit perturbed circadian rhythm with fragmented sleep before disease onset. This study was designed to evaluate the effect of a 40-Hz light flicker on circadian rhythm in an AD mouse model (APP/PS1). Locomotor rhythms recordings were conducted to examine the circadian clock rhythm in APP/PS1 mice. Molecular biology analyses, including western blot and real-time qPCR assays, were conducted to assess the changes in circadian locomotor output cycles kaput (CLOCK), brain and muscle arnt-like protein-1 (BMAL1), and period 2 (PER2). In addition to determining the direct effect of a 40-Hz light flicker on hypothalamic central clock, whole-cell voltage-clamp electrophysiology was employed to record individual neurons of suprachiasmatic nucleus (SCN) sections. The results reported herein demonstrate that a 40-Hz light flicker relieves circadian rhythm disorders in APP/PS1 mice and returns the expression levels of key players in the central circadian clock, including Clock, Bmal1, and Per2, to baseline. Moreover, the frequency of spontaneous inhibitory postsynaptic currents (sIPSCs) in SCN neurons is significantly lower in APP/PS1 mice than in the control, and the amplitude of sIPSCs is decreased. Exposure to a 40-Hz light flicker significantly increases the sIPSC frequency in SCN neurons of APP/PS1 mice, with little effect on the amplitude. However, the frequency and amplitude of spontaneous excitatory postsynaptic currents (sEPSCs) are both unaffected by a 40-Hz light flicker. The data suggest that a 40-Hz light flicker can ameliorate AD-associated circadian rhythm disorders, presenting a new type of therapeutic treatment for rhythm disorders caused by AD.

## Introduction

Alzheimer’s disease is a neurogenic disease presenting with dementia. The main pathological features of AD include the deposition of extracellular amyloid-β (Aβ) plaques and the buildup of intracellular hyperphosphorylated tau and neurofibrillary tangles (NFTs) (Khan et al., 2016). The primary symptoms of AD are severe progressive cognitive deficits, gradual memory loss, and circadian rhythm disturbances.

Circadian disturbances have been reported in approximately 45% of AD patients (Saeed and Abbott, 2017), presenting as reduced locomotor activity, fragmented sleep, sundowning syndrome, and disrupted core body temperature (Prinz et al., 1982; Satlin et al., 1995; Bliwise, 2004; Bliwise et al., 2011; Van Erum et al., 2018). Frequent nighttime awakening and excessive daytime naps greatly affect the quality of life of patients, family members, and caregivers (Vitiello et al., 1991). More importantly, circadian rhythm disturbances may represent preclinical preceding overt cognitive symptoms and memory loss (Carpenter et al., 1996; Moran et al., 2005; Vanderheyden et al., 2018). Recent studies suggest that disrupted circadian rhythms may be a principal component of the causal pathway, leading to AD pathogenesis and progression (Vanderheyden et al., 2018).

Circadian rhythm refers to the biological tendency of the body to operate in 24-h cycles. The circadian rhythm system is commonly affected in many neurodegenerative diseases, including AD, Parkinson’s disease (PD), and diffuse Lewy body (DLB) dementia (Kimoff, 1996; Dauvilliers, 2007; Deschenes and McCurry, 2009; Rolls, 2011; Saeed and Abbott, 2017). The central clock supporting circadian rhythmic behavior, including wake and sleep, is positioned in the suprachiasmatic nucleus (SCN) of the hypothalamic forebrain. As the central pacemaker, the SCN receives light information from the retina and transmits the synchronization signal to peripheral clocks in different parts of the body, such as the liver, heart, and adipose tissue, in order to regulate cellular and physiological functions (Reppert and Weaver, 2002; Panda et al., 2002; Schibler et al., 2003). SCN transplantation from younger animals can restore multiple circadian rhythm and the life span of aged animals (Hurd and Ralph, 1998; Li et al., 1998).

The rhythm’s output is presently thought to depend primarily on the transcription − translation feedback loop, which consists of clock-controlled genes and a series of clock genes. Brain and muscle arnt-like protein-1 and period 2 (PER2) are the core constituents of the transcription − translation feedback loop and are closely related to circadian rhythm maintenance. Previous studies indicate that mice lacking the *Bmal1* gene become arrhythmic when passing from a light–dark (LD) cycle to constant darkness (DD) (Bunger et al., 2000). Additionally, Per2^–/–^ mice have shown a significant interruption of the rhythm of running activity (Zheng et al., 2001). Circadian rhythm disorders can manifest in a number of ways, with the most obvious change being the sleep − wake cycle. Patients with neurodegenerative diseases often observe irregular sleep–wake cycles, and they tend to sleep multiple times within 24 h. Patients report symptoms of insomnia, difficulty falling asleep, and excessive sleepiness during the day (Mattis and Sehgal, 2016). The increase in sleepiness during the day is linked to a high risk of dementia (Merlino et al., 2010; Mattis and Sehgal, 2016).

The manifestation of circadian rhythm disorders in patients of AD is associated with the deposition of Aβ (Cedernaes et al., 2017). Circadian rhythm disorders and Aβ deposition were observed in the brain of 5ÕFAD mice, and 5ÕFAD mice are also used as an AD model (Song et al., 2015). However, there is a lack of therapy for circadian rhythm disorders caused by AD. Understanding the relationship between AD and circadian rhythm disorders is important for the improvement of AD treatment. Timely detection of sleep and circadian rhythm disorders can provide biomarkers of AD, which can be used as treatment targets or to monitor disease progression.

Light therapy has proven effective in improving sleep disturbances caused by neurodegenerative disorders, such as PD (Wu and Swaab, 2005; Videnovic et al., 2017). It has been reported that light therapy can restore melatonin production and relieve clinical circadian disturbances (Wu and Swaab, 2005). However, there are no clear guidelines available for light therapy in AD-associated circadian disturbances (Wu and Swaab, 2005; Forbes et al., 2014; van Maanen et al., 2016). In 2016, a study reported that using a 40-Hz light flicker can induce 40-Hz gamma oscillation and inhibit the production of Aβ in a mouse model of AD (Iaccarino et al., 2016). A 40-Hz light flicker activated microglia, accelerating the removal of existing amyloid deposition (Iaccarino et al., 2016; Singer et al., 2018; Martorell et al., 2019). This light treatment provides a promising therapeutic application; however, limited information is available on whether a 40-Hz light flicker could be effective in improving AD-associated circadian rhythm disorders. Our study aimed to examine how a 40-Hz light flicker affects the circadian rhythm using APP/PS1 mice, a well-characterized model of AD.

## Materials and Methods

### Animals and Housing

Adult (female, age 8 months) APP/PS1 mice were obtained from the Jackson Laboratory, with their non-transgenic wild-type (WT) littermates as control. Female WT mice were divided randomly into two groups (*n* = 10 per group): A control group (control) and a 40-Hz light flicker group (40 Hz). APP/PS1 mice were divided randomly into two groups (*n* = 10 per group): An APP/PS1 group (APP/PS1) and an APP/PS1 plus 40-Hz light flicker group (APP/PS1 + 40 Hz). Mice were housed under a 12-h light/12-h dark cycle (temperature ∼25°C; humidity ∼40%). The experiment procedures were approved by the Institutional Animal Care and Use Committee of Shenzhen University (resolution number, 2017003). All efforts were made to reduce animal suffering.

### Light Irradiation

Based on the phase response curve of light reported previously, light stimulation in the morning induces phase advances in humans (Minors et al., 1991; Bjorvatn and Pallesen, 2009; Dewan et al., 2011). In our study, the mice in the 40 Hz and APP/PS1 + 40 Hz groups were exposed to light (LED, centroid wavelength = 462.8 nm, Tc ≥ 25,000 K, time-frequency modulation frequency = 40 Hz, irradiation power density = 0.3 mW/cm^2^) at 8:00 a.m., 1 h for 30 days (Supplementary Figure S1). Mice are confined in their cages and had access to food and water *ad libitum*. A 40-Hz light flicker covers the entire cage (Supplementary Figure S1). After the last light exposure, the mice were euthanized, and their hypothalami and hippocampus tissues were collected and stored at −80°C for subsequent analysis.

### Western Blot Assays

The samples of mice were collected within 1 h after a 40-Hz light flicker. The brain was quickly and cautiously placed in cold saline (0.9%), and the hypothalamus (including the SCN) and hippocampus were dissected immediately on a cold plate surface and subsequently weighed and homogenized using a RIPA reagent (Sigma). The homogenate was subjected to 3,000 rpm of centrifugation for 30 min at 4°C. After which, the supernatant portion was collected for further analysis. BCA assay (Beyotime, Jiangsu, China) was used to determine protein concentrations. SDS/PAGE electrophoresis was used to separate the protein lysates which were then transferred onto PVDF membranes (GE Healthcare, Freiburg, Germany). Antibodies used in Western blot analysis were obtained from Abcam (Cambridge, MA, United States) unless otherwise indicated. Product numbers and antibody dilutions are indicated in parentheses. The membranes were incubated at 4°C overnight after 1 h of blocking using 5% milk. The following antibodies were used: BMAL1 (ab228594, 1:1,000), CLOCK (ab3517, 1:1,000), GAPDH (ab181602, 1:1,000), and PER2 (Millipore, AB2202, 1:1,000). Then the membrane was further incubated with a 1:5,000 HRP-conjugated goat anti-rabbit IgG in Tris–buffered saline Tween-20 (TBST) for 1 h (ab6721). The target protein was visualized using Clarity ECL Western Blotting Substrate (Bio-Rad, Hercules, CA, United States) and was quantified through densitometry with the Bio-Rad Quantity One software.

### RNA Isolation and Real-Time qPCR

An RNA extraction kit (MiniBEST Universal RNA Extraction Kit, TaKaRa, Dalian, China) was used to extract the total mRNA. The PrimeScript RT Master Mix reverse-transcription kit was used to reverse RNA into cDNA, and this was after evaluation of purity of the extracted RNA. Real-time PCR with Power SYBR Green PCR Master Mix (Bio-Rad, Hercules, CA, United States) on an Analytik Jena QPCR System was used to determine the relative gene expression. The comparative threshold cycle (ΔΔCt) was used for the quantification of the relative expression of mRNA. The housekeeping gene (GAPDH) was used as control. The specific primers for gene expression analysis are shown in Table 1.

**TABLE 1:** Primer sequences used for quantitative RT-PCR analysis.

**Gene**	**Forward (5′–3′)**	**Reverse (5′–3′)**
mBmal1	5′-ACAATGAGCC AGACAACG-3′	5′-TTCCCATCTA TTGCGTGT-3′
mPer2	5′-CACTTGCCTC CGAAATAA-3′	5’-ACTACTGCCTCT GGACTGG-3′
mClock	5′-TCACCACGTTCA CTCAGGACA-3′	5’-AAGGATTCCC ATGGAGCAA-3′
mGAPDH	5′-CTTGTGCAG TGCCAGCC-3′	5’-GCCCAATACG GCCAAATCC-3′

### Locomotor Rhythms Recording

Adult (female, age 8 months) APP/PS1 mice (*n* = 3) and their non-transgenic WT littermates (*n* = 3) were used for recording. Mice were individually housed within PhenoTyper 3000 cages (installed with a light flicker), and they were allowed *ad libitum* access to food and water. EthoVision XT is the applied video tracking software that tracks and analyzes the behavior, movement, and activity of any animal. EthoVision XT V14 was used to record and analyze locomotor behavior. Spontaneous locomotor activity was defined as the moving distance per unit time (3 min). Mice were videotaped for 3 days, and the locomotor rhythms recordings were analyzed as control and APP/PS1. For the following 30 days (due to the limitation of the recording time of the system, the data of 12 days are displayed), mice were given 40-Hz light flicker treatment, and their locomotor rhythms recordings were analyzed as 40 Hz and APP/PS1 + 40 Hz.

### Measurement of Body Weight, Heart Rate, and Fasting Blood Glucose Level

Mice were weighted before and after 30 days of 40-Hz light flicker treatment. The heart rate of anesthetized mice was measured by a Vevo 2100 system (VisualSonics, Toronto, Canada). Fasting blood glucose level was detected from the tail vein after 8 h of fasting using an automated glucometer (GM100; Bionime GmbH, Berneck, Switzerland).

### Visual Evoked Potential

Mice were dark-adapted, their pupils dilated, and anesthetized by intraperitoneal injection with ketamine (75 mg/kg) and xylazine (10 mg/kg). Visual evoked potential was performed using a RETI-port/scan 21 recorder (Roland Consult, Wiesbaden, Germany) according to the manufacturer’s instructions.

### Necropsy and Slice Preparation

The mice were first anesthetized with isoflurane. To perform craniotomy, the brain was quickly removed, followed by isolation of the tissue block containing SCN. The tissue block was then transferred to a cutting solution (ice-cold) containing (in mM) 30 CaCl, 194 sucrose, 1 MgCl_2_, 4.5 KCl, 26 NaHCO_3_, and 1.2 NaH_2_PO_4_, aerated by a mix of 5% CO_2_ and 95% O_2_ gases. A ceramic blade (Camden Instruments Ltd.) joined to a vibratome tissue slicer (VT1200 S, Leica Biosystems, Wetzlar, Germany) was used to obtain coronal sections that were 300 μm thick. The slices were then equilibrated in an artificial cerebrospinal fluid (CSF) at 33°C for 1 h and then kept at room temperature.

### Slice Electrophysiology

The brain sections were moved to a recording chamber attached to an upright microscope stage and perfused continuously (after stabilization using an overlying platinum ring) to maintain the solution at 28−32°C. Infrared optics with a 40× water immersion objective was used to identify a single neuron in the SCN, and the image was displayed on a computer monitor. The pipettes were removed from a borosilicate glass capillary (1.5-mm OD, 0.86-mm ID, Sutter Instruments Co., Novato, CA, United States) and filled with potassium chloride or potassium gluconate (KGluc). KCl-based intracellular solutions (290–295 mOsmol, pH 7.25) were used for inhibitory synaptic transmission and excitatory recordings. The KGluc solution contained (in mM) K-110 gluconate, 20 KCl, 10 HEPES, 0.3 Na_2_GTP, 4 MgATP, and 20 KCl, and the KCl solution contained (in mM) 2 MgCl_2_, 125 KCl, 2.8 NaCl, 2 MgATP, 0.3 Na_2_GTP, 10 HEPES, and 0.6 EGTA. When recording spontaneous excitatory synaptic currents (sEPSCs), bicuculline (10 μM) was added to aCSF. To record spontaneous inhibitory postsynaptic currents (sIPSCs), NBQX (5 μM) and D-APV (50 μM) were added to aCSF. A MultiClamp 700B amplifier (Molecular Devices, Foster City, CA) was used to record the currents. Whole-cell membrane currents were digitized at 20 kHz and filtered at 2 kHz. The pCLAMP software (Molecular Devices, Sunnyvale, CA, United States) was used for analysis.

### Statistical Analyses

Data were reported as mean ± SD of no less than three separate experiments. Statistical analysis was conducted by one-way analysis of variance (ANOVA) and with Tukey’s *post hoc* test. An least significant difference (LSD) *t*-test was used for comparison between groups. Statistically significant differences between groups were defined at *p* < 0.05. All of the analyses were completed with GraphPad Prism software (San Diego, CA, United States).

## Results

### A 40-Hz Light Flicker Alleviates the Circadian Rhythm Disorders in APP/PS1 Mice

**FIGURE 1:**
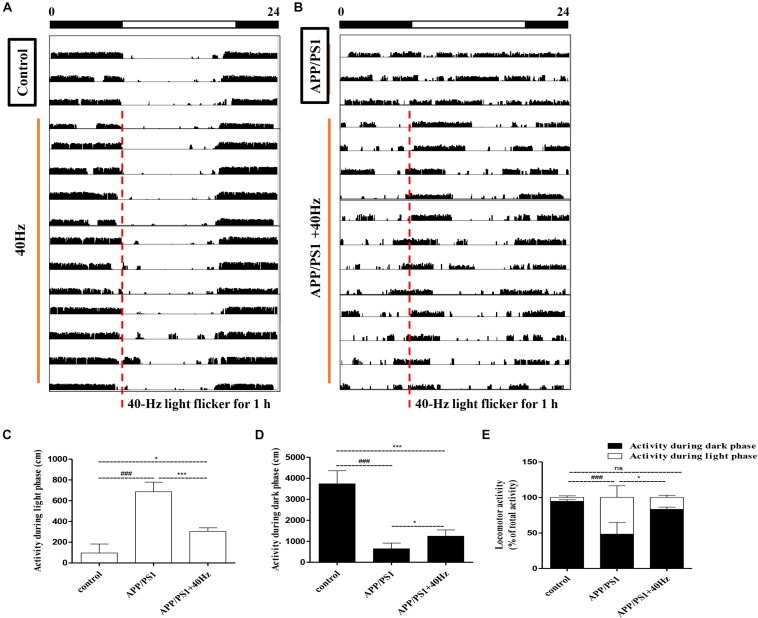
Influence of 40-Hz light flicker on circadian rhythm disorder in APP/PS1 mice. **(A,B)** Representative average locomotor activity records. Locomotor activity was defined as the moving distance per unit time (3 min). The red line represents 1-h of flickering light exposure. The black and white bars on the top indicate dark and light periods, respectively. **(C)** Activity during the light phase (cm). **(D)** Activity during the dark phase (cm). **(E)** Percentage of the locomotor activity in the dark (12 h) to total activity (24 h). ^###^*p* < 0.001 significantly different from the control group; *p < 0.005, ***p < 0.001, significantly different from the APP/PS1 group.

Evaluations of spontaneous motor activity patterns under standard light and dark conditions (LD12:12) were performed on WT and APP/PS1 mice to determine circadian rhythm function at baseline. In our study, the mice were exposed to a 40-Hz light flicker at 8 a.m. for 1 h in a special cage (Supplementary Figure S1). Under the condition of LD12:12, control group mice were more active in the dark than in the light, and occasionally, there was activity in the light phase (Figures 1A–D) consistent with mice being a nocturnal species (96 ± 86 cm in the light and 3,734 ± 622 cm in the dark). The daily exercise rhythm of the LD12:12 photoperiod showed stable diurnal activity.

However, APP/PS1 mice lose the irregular daily activity–rest cycle, with their rest phase being much more fragmented compared to that of control group mice (Figures 1B–D). The results are consistent with those of a previous study (Sethi et al., 2015), showing that 5XFAD mice exhibit alterations in their sleep–wake patterns. Spontaneous locomotor activities during the light phase were significantly prolonged; by contrast, activities at dark were significantly reduced compare to those of control group mice (686 ± 91 cm in the light and 644 ± 268 cm in the dark). Interestingly, increased regular locomotor activity was observed after a 40-Hz light flicker in AD mice (Figure 1B). And spontaneous locomotor activities in APP/PS1 + 40 Hz group mice were significantly decreased in the light and increased in the dark compared to those of APP/PS1 group mice (304 ± 33 cm in the light and 1,242 ± 298 cm in the dark). Percentages of the locomotor activity in the dark (12 h) to total activity (24 h) in the control group, APP/PS1 group, and APP/PS1 + 40 Hz group were 94 ± 2, 48 ± 16, and 83 ± 3, respectively. Compared to that in the APP/PS1 group, the percentage of the locomotor activity in the dark (12 h) to total activity (24 h) was significantly increased in the APP/PS1 + 40 Hz group (Figure 1E). These results suggest that the fragmented rest phase of AD mice has been restored greatly and that a 40-Hz light flicker alleviates circadian rhythm disorders in APP/PS1 mice.

### A 40-Hz Light Flicker Has Little Effect on the Normal Physiological Status of Mice

**FIGURE 2:**
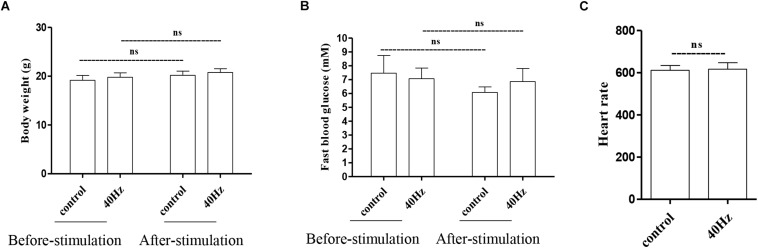
Effect of 40-Hz light flicker on normal physiological status of mice. Body weight **(A)**, heart rate **(B)**, and the level of fasting blood glucose **(C)** before and after 30 days of light treatment. ns, no significant difference.

We assessed the effect of a 40-Hz light flicker on basic physiological parameters, including body weight, heart rate, and fasting blood glucose levels. The results showed that there was no significant difference in mouse weight before (20.75 ± 0.77 g) and after (19.7 ± 0.9 g) a 40-Hz light flicker (Figure 2A). Similarly, there were no significant differences in the fasting blood glucose levels (7.0 ± 0.7 and 6.8 ± 0.9 mM) and heart rate (611.6 ± 22.6 and 616.6 + 30.8) before and after a 40-Hz light flicker (Figures 2B,C). Our results suggested that a 40-Hz light flicker did not affect the body weight, heart rate, and blood glucose levels of the mice.

### A 40-Hz Light Flicker Has Little Impact on Retinal Electrophysiology in Mice

**FIGURE 3:**
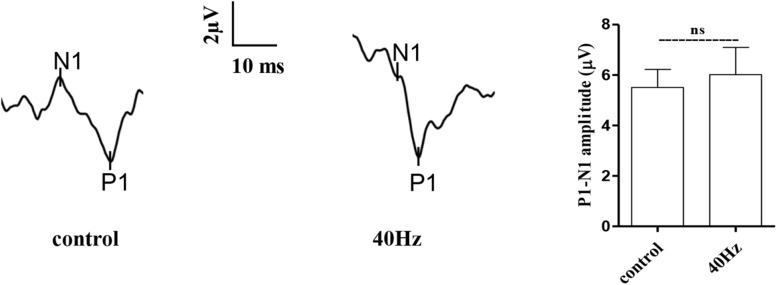
Effect of 40-Hz light flicker on retinal electrophysiology in mice. Representative waveforms of visual evoked potential (VEP) of eyes in mice before and after 30 days of light treatment. The differences in peak amplitude (N1–P1) were quantified. Data are expressed as means ± SEM. *n* = 5 eyes per group. For each eye, data from three independent curvilinear diagrams were averaged, and the mean of five eyes was used as the representative value for each group. ns, no significant difference.

The light that enters the hypothalamus regulates the central circadian clock and synchronizes the peripheral clocks. In this study, we hypothesize that a 40-Hz light flicker might have an impact on retinal function. The physiological function of retinal ganglion cells can be evaluated by examining visual evoked potentials (VEPs). VEPs detect light conduction of retinal ganglion cells to the visual center, and the peak amplitude difference (N1–P1) reflects the visual response. Interestingly, we did not observe significant changes in the amplitude of P1–N1 when comparing the control group (5.5 ± 0.7 μV) with the 40-Hz light flicker group (6.0 ± 1 μV) (Figure 3). A 40-Hz light flicker did not disturb the mouse retinal function significantly. Our results suggest that short exposure to a 40-Hz light flicker had little impact on basic physiological functions.

### A 40-Hz Light Flicker Induced Gamma Power of Cortical ECoG and Reduced Aβ and Tau Production in the Hippocampus of APP/PS1 Mice

**FIGURE 4:**
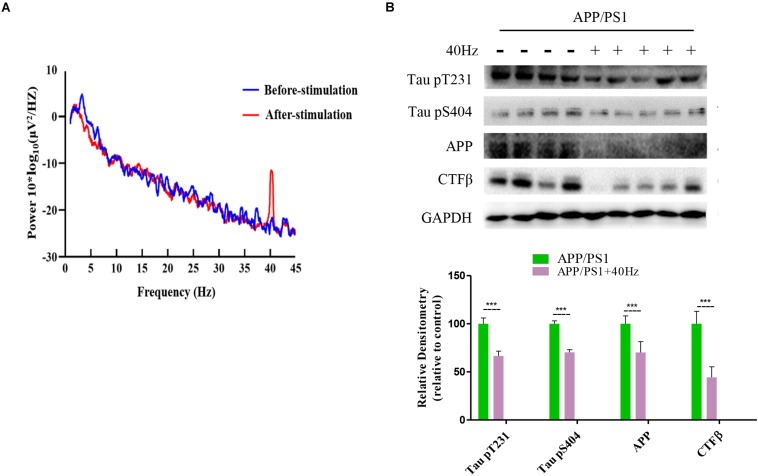
A 40-Hz light flicker induced gamma power of cortical ECoG and reduced Aβ and Tau production in the hippocampus of APP/PS1 mice. **(A)** Local field potential trace of visual cortex before and during 40-Hz light flicker. **(B)** Protein expression levels of Tau pT231, Tau pS404, APP, and CTFβ of mice hippocampus were determined after the last 40-Hz light flicker by five independent western blots. ***p < 0.001, significantly different from the APP/PS1 group.

It was reported that a 40-Hz light flicker improved altered gamma in multiple brain regions and inhibited the production of Aβ (Iaccarino et al., 2016). We tested in APP/PS1 mice and observed that a 40-Hz light flicker induced the gamma in the visual cortex (Figure 4A). We next assessed the effect of a 40-Hz light flicker on APP and tau protein levels in the hippocampus. The results show that 40-Hz light flicker treatment significantly decreased protein expressions of APP and phosphorylated tau in comparison with those of APP/PS1 mice (100 ± 8.13, 100 ± 6.04, and 100 ± 3.04 in the APP/PS1 group and 70.14 ± 11.26, 66.56 ± 4.99, and 70.26 ± 2.76 in APP/PS1 + 40 Hz group, respectively) (Figure 4B). The Aβ is processed by β-secretase cleavage of APP to produce β-CTF; we also found that β-CTF was significantly reduced with a 40-Hz light flicker compared to the APP/PS1 group (100 ± 12.96 in the APP/PS1 group and 44.25 ± 11.02 in the APP/PS1 + 40 Hz group) (Figure 4B).

### A 40-Hz Light Flicker Restores the Expression Levels of Key Central Clock Proteins in the SCN of APP/PS1 Mice

**FIGURE 5:**
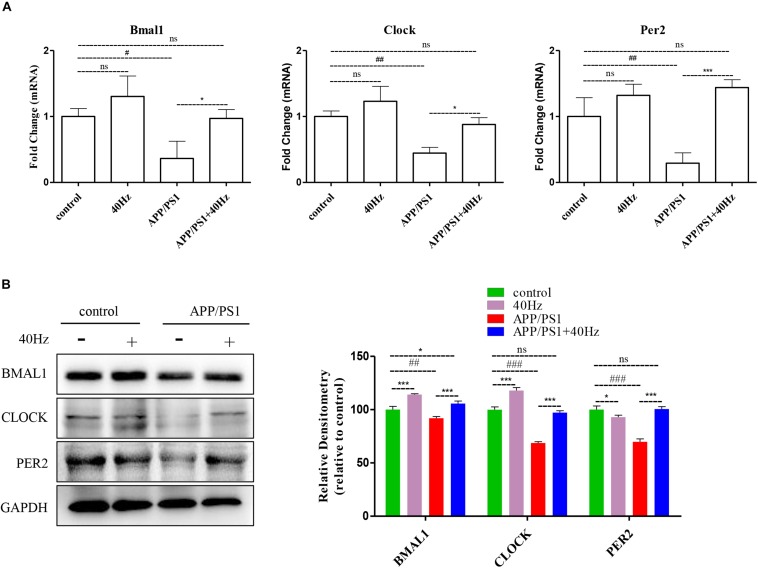
A 40-Hz light flicker restores the expression levels of key players of central clock in the SCN of APP/PS1 mice. **(A)** mRNA expression of Bmal1, Clock, and Per2 in the SCN from control and APP/PSI mice with or without light exposure (*n* = 6). **(B)** Representative western blot showing protein expression levels of BMAL1, CLOCK, and PER2 of hypothalamus of mice with or without light exposure. Quantification was determined by five independent experiments. ^#^*p* < 0.005, ^##^*p* < 0.01, ^###^*p* < 0.001 significantly different from the control group; *p < 0.005, ***p < 0.001, significantly different from the APP/PS1 group.

A 40-Hz light flicker improved the circadian rhythm behavior of APP/PS1 mice. This finding suggests that a 40-Hz light flicker could affect and change the physiological function of the central circadian pacemaker, the SCN. To test this hypothesis, we examined the effects of daily 1-h exposure to a 40-Hz light flicker on clock gene expression in the SCN of APP/PS1 mice. We found that the hypothalami of APP/PS1 mice have a significant reduction of the Bmal1, Clock, and Per2 mRNA levels compared to those of control mice (1 ± 0.13, 1 ± 0.08, and 1 ± 0.28 in the control group and 0.36 ± 0.26, 0.44 ± 0.08, and 0.29 ± 0.15 in the APP/PS1 group, respectively) (Figure 5A). It is interesting that the mRNA levels (0.97 ± 0.13, 0.88 ± 0.10, and 1.4 ± 0.11, respectively) were partly restored with a 40-Hz light flicker (Figure 5A). Single 40-Hz light flicker administration showed no effects on the mRNA levels of Bmal1 (1.30 ± 0.30), Clock (1.23 ± 0.22), and Per2 (1.32 ± 0.16) compared with the control group. To further evaluate the protein expression levels of BMAL1, CLOCK, and PER2, we performed western blotting analysis. A significant reduction in the expression of BMAL1 (91.89 ± 1.7), CLOCK (68.73 ± 1.2), and PER2 (69.84 ± 2.77) proteins was also found in APP/PS1 mice (Figure 5B). In contrast, 40-Hz light flicker treatment significantly increased protein expressions of BMAL1 (105.72 ± 2.2), CLOCK (97.2 ± 1.7), and PER2 (100.5 ± 2.31) in comparison with APP/PS1 mice (Figure 5B). These results indicate that a 40-Hz light flicker regulated the Bmal1, Clock, and Per2 protein expressions in the hypothalami of APP/PS1 mice.

### A 40-Hz Light Flicker Stimulated the Amplitude of sIPSC and Frequency of sEPSC on SCN Neurons in APP/PS1 Mice

**FIGURE 6:**
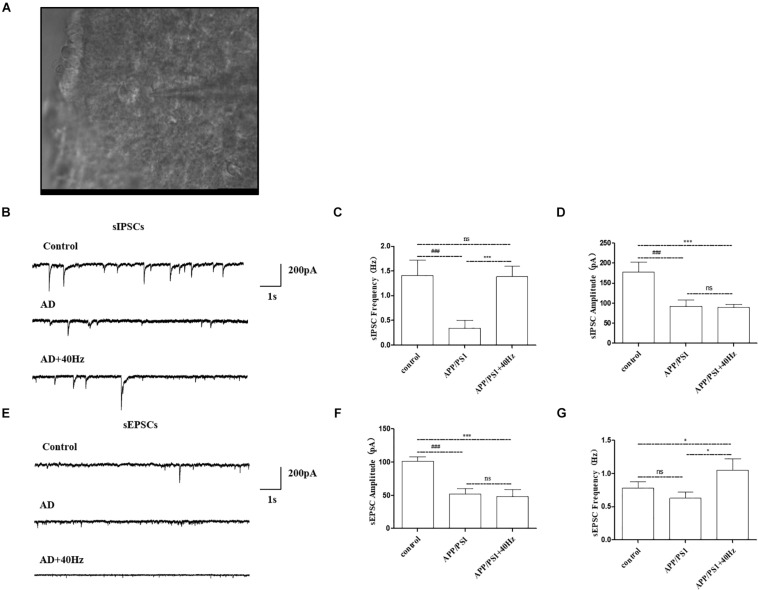
Effect of 40-Hz light flicker on SCN neurons in APP/PS1 mice. **(A)** Infrared images of the neurons recorded at high power (40 ×) in the SCN. **(B)** The representative traces of spontaneous inhibitory postsynaptic currents (sIPSCs). **(C)** Changes in the frequency of sIPSCs in SCN neurons of each group of mice. **(D)** Changes in sIPSC amplitude in SCN neurons of each group of mice. **(E)** The representative traces of spontaneous excitatory postsynaptic currents (sEPSCs). **(F)** Changes in the frequency of sEPSCs in SCN neurons of each group of mice. **(G)** Changes in sEPSC amplitude in SCN neurons of each group of mice. All of the data in panels **(B–G)** are presented in the natural space as mean ± SD. ^###^*p* < 0.001, significantly different from the control group; *p < 0.005, ***p < 0.001, significantly different from the APP/PS1 group. ns, no significant difference.

Given that expression of key circadian genes was changed in the hypothalami of APP/PS1 mice exposed to a 40-Hz light flicker, we monitored the physiological function of SCN cells. Whole-cell voltage-clamp electrophysiological recordings were performed in individual central clock neurons from acutely prepared coronal SCN slices from APP/PS1 mice receiving a 40-Hz light flicker and from WT mice (Figure 6A). The membrane resistance, including both sIPSCs and sEPSCs, was recorded as the response variable.

Notably, AD mice have perturbed physiology of the central clock neuron with less spontaneous inhibitory postsynaptic firing rate (Figure 6B). The frequency of sIPSCs in central clock neurons was significantly decreased with a lower firing amplitude in APP/PS1 mice compared with that in the control group (1.4 ± 0.3 Hz and 177.9 ± 25.2 pA in the control group and 0.3 ± 0.1 Hz and 92.7 ± 15.9 pA in the APP/PS1 group). Interestingly, a 40-Hz light flicker restored the frequency of sIPSCs in SCN neurons significantly in APP/PS1 mice but had little effects on firing amplitude (1.3 ± 0.2 Hz and 89.3 ± 8.2 pA in the APP/PS1 + 40 Hz group) (Figures 6C,D). In contrast, SCN neurons of APP/PS1 mice (51.8 ± 8.6 pA and 0.6 ± 0.09 Hz) exhibited reduced sEPSC amplitude with no significant change in firing frequency compared with those of control animals (101.5 ± 6.4 pA and 0.7 ± 0.09 Hz) (Figures 6E,F). However, a 40-Hz light flicker stimulated the sEPSC frequency (1.0 ± 0.09 Hz) without affecting the neuronal firing amplitude (48.2 ± 10.6 pA) (Figures 6F,G). This suggests that the basal inhibitory tone was increased in the central clock neurons of APP/PS1 mice.

## Discussion

Abnormal deposition of Aβ, hyperphosphorylation of tau protein, and loss of neuronal cells are typical pathological changes observed in AD patients (Goedert and Spillantini, 2006). Managing AD is currently in a limited pharmacotherapy stage. Currently, the AD drugs used in clinical therapy are limited to acetylcholinesterase (AChE) inhibitors and *N*-methyl-D-aspartate (NMDA) receptor antagonists (Bachurin et al., 2017). However, these drugs can only relieve certain symptoms without altering or delaying the progression of AD. Therefore, new AD therapies are urgently needed.

Recently, there has been experimental evidence that 40-Hz light flickering could cure AD by inducing 40-Hz gamma oscillations and inhibiting the production of Aβ in an AD mouse model (Iaccarino et al., 2016). This effect is only observed for light flickered at the frequency of 40 Hz. Constant light and 20-Hz, 80-Hz, or random light flickers exhibit insignificant effects on Aβ levels (Iaccarino et al., 2016). In this study, we show that the 40-Hz light flicker ameliorates circadian rhythm disorder in the APP/PS1 AD mouse model by reducing the deposition of Aβ in the hypothalamus and increasing the rhythmic expression of clock proteins, such as BMAL1, CLOCK, and PER2. After 30 days of 40-Hz-flickering light treatment, no adverse effects on mouse body weight, blood glucose level, heart rate, and biological rhythm were observed.

Previously, it had been shown that Aβ deposition can directly drive impaired sleep in animals (Cedernaes et al., 2017). Based on the results of the wheel-running activity assay, mice injected with Aβ31-35 in the hippocampus exhibit obvious circadian rhythm abnormalities with significantly longer free-running periods than those in the control (Wang et al., 2016). Herein, we show that 40-Hz light flickering can improve diurnal activity in mice. Specifically, pretreatment with 40-Hz-flickered light alleviates the irregular daily activity/rest cycle in APP/PS1 mice suffering from circadian rhythm disorders and restores the ratio of nocturnal to total activity. This indicates that the fragmented rest phase in AD mice is greatly restored by 40-Hz light flicker treatment, and this treatment effectively alleviates circadian rhythm disorders in APP/PS1 mice.

The term “circadian rhythm” is generally used to describe a variety of periodically changing physiological and biochemical activities in different organisms (Chang and Guarente, 2013). The circadian regulation is accomplished through the central oscillator in the SCN (Chang and Guarente, 2013). SCN is the mammalian circadian rhythm center that integrates external rhythmic stimuli, such as external light, environmental temperature, and food, in a continuous circadian rhythm of approximately 24 h, thereby regulating the diurnal variation of physiological activities (Takahashi, 2017). The daily rhythm of SCN is regulated by a transcription and translation feedback loop system involving clock genes like *Clock*, *Bmal1*, and *Per2*. As shown in Figure 5, the expressions of these genes in the hypothalami of APP/PS1 mice are significantly decreased, which suggests that Aβ deposition leads to major circadian rhythm abnormalities in mice. As expected, a 40-Hz light flicker increases the expression of Bmal1, Clock, and Per2 that had been suppressed by Aβ deposition. Therefore, a 40-Hz light flicker eliminates circadian rhythm disorders in APP/PS1 mice.

The retina receives environmental light and transmits it to the SCN by the retinohypothalamic tract. Herein, we investigated whether 40-Hz light flickering could affect the output signal transmitted to the SCN from the retina. Interestingly, our results show that long-term daily exposure to 40-Hz-flickered light alters the AD-associated activity/rest pattern without significantly changing the retinal visual ability. This suggests that the retinal signals induced by flashing light are transient; however, they have a cumulative effect on clock neurons. In addition, the SCN innervates other brain regions with electrical signals, resulting in the encoding of circadian rhythm information, such as rhythmic secretion of melatonin from the Pineal gland (Teclemariam-Mesbah et al., 1999). Therefore, the electrophysiological data of SCN neurons may offer some insight into the mechanism underlying the restoration of circadian rhythm by a 40-Hz light flicker. Neurons in the SCN of APP/PS mice exhibit reduced spontaneous firing with lower frequency and amplitude than those of WT animals. A 40-Hz light flicker increases the firing frequency with no effect on amplitude. Elevated spontaneous firing of cells is expected to increase locomotor activity and suppress sleep. Indeed, in the hours following 40-Hz light flicker, enhanced locomotor activity of APP/PS1 animals was observed. This might be related to the circadian regulation of ion channel, intracellular trafficking, and other proteins that affect membrane potential. However, the association between the recalibrated central circadian clock network and the electrophysiological status of SCN neuron should be further investigated, along with the relationship between increased neuronal firing rate and elevated gamma waves induced by 40-Hz light flickering.

At present, we do not know whether the restored electrophysiology of SCN neurons is due to the direct effect of light stimulation or reduced amyloid deposition in other parts of the brain. However, a recent study shows that the effect of 40-Hz light flicker in reducing the amyloid level is mediated by enhanced amyloid endocytosis in the microglia (Adaikkan et al., 2019). Generally, the function of the microglia is regulated by the sleep–wake cycle, and the injury response of these cells is reduced in the awake brain (Griffin et al., 2019; Stowell et al., 2019). We speculate that a 40-Hz light flicker directly stimulates central clock neurons, improves circadian rhythm, restores activity–rest cycle, and boosts microglial amyloid surveillance in AD animals. To confirm this hypothesis, investigations of the microglia function in SCN-damaged AD mice are needed. Overall, the results reported herein and those published previously indicate that the restoration of the circadian rhythm is necessary for the treatment of neurodegenerative diseases and that light stimulation constitutes a promising therapeutic technique.

Based on a previous study, the AD mice tested herein were subjected to daily treatment (1 h/day) with 40-Hz flashing light for 30 days (Iaccarino et al., 2016). In the future, the effect of 40-Hz light flickering on human AD models should be studied in order to establish clinical applications of the proposed light therapy. The timing and duration of light exposure are key variables affecting therapeutic efficiency, and thus, they should be optimized and controlled, especially considering that inappropriately timed light will likely worsen the symptoms of circadian rhythm disorder (Bjorvatn and Pallesen, 2009). It should be noted that the 40-Hz light flicker therapy experiments performed herein were scheduled at 8:00 a.m., the beginning of the natural light phase. However, little is known about the time or duration of phototherapy required to clinically treat AD. Further study should be carried to test whether a similar effect could be observed in humans considering that mice are nocturnal animals. Timed exposure is inconvenient, and as with any lifestyle change, compliance can be a major issue. Measurements of core body temperature or endogenous melatonin rhythms allow for the assessment of the circadian phase, which in turn permits the time scheduling needed to increase the efficiency of clinical AD treatment (Bjorvatn and Pallesen, 2009). In the future, we shall study the effect of timing of 40-Hz light stimulation on melatonin secretion. The effects of the spectral characteristics of light, such as wavelength, frequency, intensity, and temperature, should also be tested.

## Conclusion

Our research demonstrated that a 40-Hz light flicker (40 Hz, 462.8 nm, at 8:00 a.m., 1-h) for 30 days improves fragmented locomotor activity–rest cycle in APP/PS1 mice and reduces the deposition of Aβ. It also alters the expression of key circadian proteins and restores the electrophysiological changes in the hypothalamus of AD mice. This indicates that a 40-Hz light flicker might be a potential candidate to treat the rhythm disorder in AD.

## Data Availability Statement

The datasets generated for this study are available on request to the corresponding author.

## Ethics Statement

The animal study was reviewed and approved by the Institutional Animal Care and Use Committee of Shenzhen University.

## Author Contributions

YYa, YYi, and YZ are the primary investigators in this study. QD, WZ, HZ, and ZL participated in part in the animal experiments. SZ participated in part in data analysis. YZ designed this study and wrote the manuscript as corresponding author. JM prepared all the light irradiation devices needed for the experiment.

## Conflict of Interest

The authors declare that the research was conducted in the absence of any commercial or financial relationships that could be construed as a potential conflict of interest.

## Supplementary Material

FIGURE S1 Parameters of light and cage used in the experiment. **(A)** Centroid wavelength = 462.8 nm, color temperature Tc ≥ 25,000 K, flashing frequency = 40 Hz. **(B)** Light exposure experimental schematic. Intensity of light is 3 mW/cm2.

## Abbreviations

AD: Alzheimer’s disease
Bmal1: brain and muscle arnt-like protein-1
Clock: circadian locomotor output cycles kaput
Per: period
SCN: suprachiasmatic nuclei.
