# Supplementary figures and images for: Non-invasive 40-Hz Light Flicker Ameliorates Alzheimer’s-Associated Rhythm Disorder via Regulating Central Circadian Clock in Mice
Source: Front Physiol. 2020 Apr 24;11:294. doi: 10.3389/fphys.2020.00294 (PMC7193101; doi:10.3389/fphys.2020.00294)

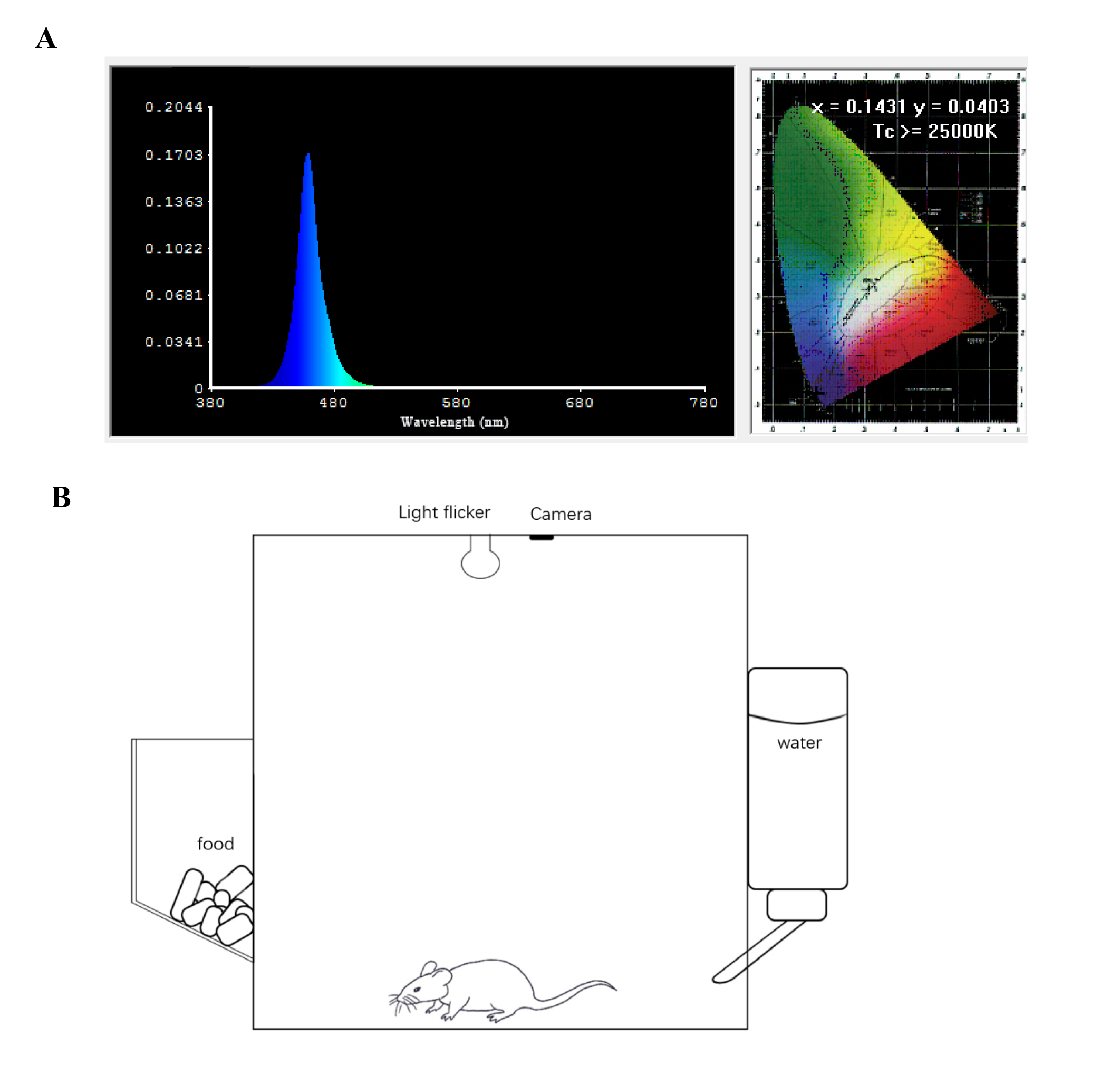

Supplement: FIGURE S1 — Parameters of light and cage used in the experiment. (A) Centroid wavelength = 462.8 nm, color temperature Tc ≥ 25,000 K, flashing frequency = 40 Hz. (B) Light exposure experimental schematic. Intensity of light is 3 mW/cm2. [file Image_1.TIF]
